# Comparing listeriosis risks in at-risk populations using a user-friendly quantitative microbial risk assessment tool and epidemiological data

**DOI:** 10.1017/S0950268816000327

**Published:** 2016-03-28

**Authors:** L. E. FALK, K. A. FADER, D. S. CUI, S. C. TOTTON, A. M. FAZIL, A. M. LAMMERDING, B. A. SMITH

**Affiliations:** 1 Public Health Agency of Canada, Guelph, Ontario, Canada; 2 University of Toronto, Toronto, Ontario, Canada; 3 University of Guelph, Guelph, Ontario, Canada; 4 University of Waterloo, Waterloo, Ontario, Canada

**Keywords:** At-risk populations, food safety, *Listeria*, risk factors, vulnerability

## Abstract

Although infection by the pathogenic bacterium *Listeria monocytogenes* is relatively rare, consequences can be severe, with a high case-fatality rate in vulnerable populations. A quantitative, probabilistic risk assessment tool was developed to compare estimates of the number of invasive listeriosis cases in vulnerable Canadian subpopulations given consumption of contaminated ready-to-eat delicatessen meats and hot dogs, under various user-defined scenarios. The model incorporates variability and uncertainty through Monte Carlo simulation. Processes considered within the model include cross-contamination, growth, risk factor prevalence, subpopulation susceptibilities, and thermal inactivation. Hypothetical contamination events were simulated. Results demonstrated varying risk depending on the consumer risk factors and implicated product (turkey delicatessen meat without growth inhibitors ranked highest for this scenario). The majority (80%) of listeriosis cases were predicted in at-risk subpopulations comprising only 20% of the total Canadian population, with the greatest number of predicted cases in the subpopulation with dialysis and/or liver disease. This tool can be used to simulate conditions and outcomes under different scenarios, such as a contamination event and/or outbreak, to inform public health interventions.

## Introduction

*Listeria monocytogenes* causes few cases of foodborne illness relative to other pathogens [1], but infection can lead to mortality, particularly in newborns and immunocompromised patients [2–4]. *L. monocytogenes* infection is the third leading cause of death attributed to foodborne illness in the United States [4]. Clinical signs of invasive listeriosis include meningitis, encephalitis, premature labour, miscarriage, abortion, and stillbirth [2, 5].

Some ready-to-eat (RTE) delicatessen meats that have not been dried or salted, such as ham, roast beef, and turkey breast, and hot dogs (frankfurters), can support the growth of *L. monocytogenes* at refrigeration temperatures. A 2008 outbreak of invasive listeriosis in Canada involving RTE meats resulted in 57 illnesses and 22 deaths [6]. Following this outbreak, the ‘Policy on *L. monocytogenes* in Ready-To-Eat (RTE) Foods’ was updated to improve control in high-risk foods [7]. Nevertheless, there is a need to evaluate potential risks to vulnerable populations as a preventive measure, and in the case of a future confirmed or suspected contamination event.

Quantitative microbial risk assessment (QMRA) is a predictive method that can be used to provide estimates of the number of human illnesses resulting from consumption of pathogens in food [8]. When pathogenic bacteria such as *L. monocytogenes* are detected in a food product or environment at a manufacturing or retail facility, it is sometimes necessary to estimate potential risks to consumers within a short time-frame. Performing a detailed QMRA is highly resource-intensive and often difficult to accomplish considering factors such as availability of information and time sensitivity. In some cases, conditions are more amenable to a simplified model [9]. An easy-to-use, pre-populated model would be more practical in scenarios where time-sensitive information is required, or modelling expertise is limited or unavailable. It could be used to inform health officials on relative risks under different scenarios of contamination levels and prevalence, either in a confirmed or suspected contamination scenario or during ‘peacetime’ (when no known outbreak is suspected) to explore potential future scenarios and intervention efforts.

Integration of regional prevalence of risk factors for listeriosis into a user-friendly model would allow for comparison of risks in regions and vulnerable subpopulations. Dose-response models that incorporate *L. monocytogenes* strain variability have recently been developed for selected populations vulnerable to listeriosis [10], and could be integrated with demographic information to derive population-level estimates of risk. Ross *et al*. [11] have estimated the prevalence of several at-risk subpopulations in Australia, but similar estimates have not yet been compiled for Canada.

The study objective was to develop a user-friendly QMRA model consisting of probabilistic inputs to produce estimates of the predicted number of cases of invasive listeriosis associated with the consumption of pre-packaged RTE delicatessen meats or hot dogs contaminated with *L. monocytogenes*. The goal was to create a stand-alone model to be used by public health officials, if necessary, but also readily modifiable by risk analysts if required.

## Materials and Methods

### Model overview

**Fig. 1.**
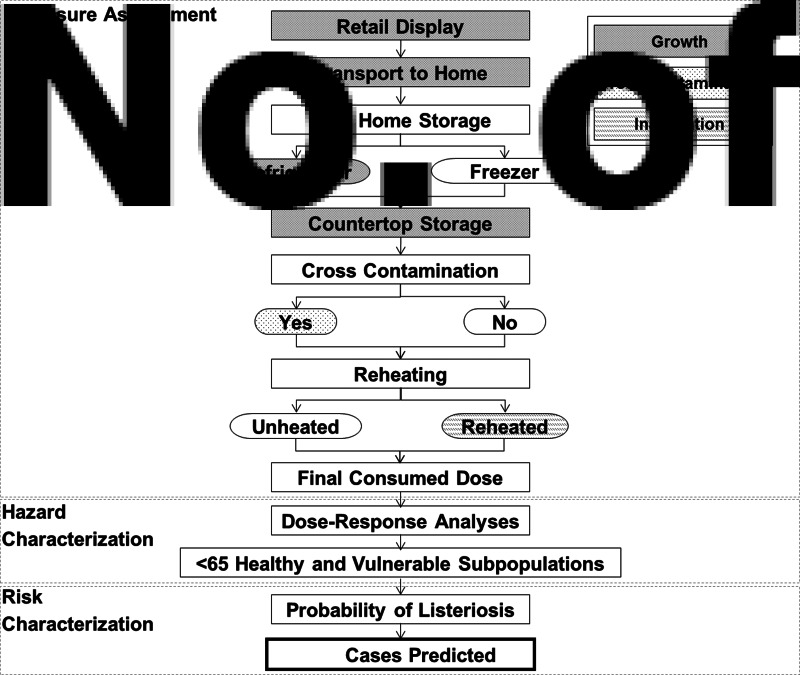
Conceptual risk assessment model for *L. monocytogenes* in ready-to-eat delicatessen meats and hot dogs.

The model produces estimates of the risk of listeriosis as a probability density function for the total number of cases predicted to occur. The median number of cases are estimated in the total population and in each of ten vulnerable subpopulations. Treatment of the product is considered from retail display to consumption. There are three main components of the model: exposure assessment, hazard characterization, and risk characterization (Fig. 1).

Processes considered within the exposure assessment component include: (i) growth during retail display, transport to home, home storage, and ambient counter-top storage, (ii) cross-contamination during kitchen preparation, and (iii) inactivation during reheating. The number of potentially contaminated portions at consumption is divided into nine categories based on contamination at retail (yes/no), type of cold storage (refrigerator/freezer), cross-contamination (yes/no), and reheating (reheated/unheated). The consumed doses are calculated for each contaminated portion category. Portions are distributed in healthy and at-risk subpopulations based on the prevalence of risk factors within each province or territory in Canada, or Canada-wide, depending on the location selected. Therefore, the proportion of product consumed by each vulnerable subpopulation is equivalent to the respective risk factor prevalence in the region.

The hazard characterization component is used to derive subpopulation-specific dose-response models. These are integrated with the results of the exposure assessment to estimate the probability of listeriosis per portion for each subpopulation of interest. Finally, based on the number of portions consumed by each subpopulation, the number of listeriosis cases is predicted in the risk characterization component (Fig. 1).

The model was implemented in @Risk 6·3 (Palisade Corp., USA), an add-in for Microsoft Office Excel (Microsoft Corp., USA), and can be obtained by request from the corresponding author. Monte Carlo analysis with Latin Hypercube Sampling is used throughout the simulation process. The model is divided into six worksheets. Users can operate the model through the Model Input and Summary sheet, where basic information must be entered, and summary results are displayed. The user must provide, at a minimum, the type of food product (RTE beef, ham, or turkey delicatessen meat, or hot dogs, with or without growth inhibitors), the number of portions potentially contaminated (e.g. the number of servings produced in one batch or set of batches at a processing facility), the prevalence and initial contamination level of the product at retail, and the geographical location (Canada-wide, or by province or territory) where the food is distributed and consumed. Once the user has entered the minimum required data, the simulation can be run. The model presents the calculated risk as a probability density function for the total number of cases predicted to occur from the potentially contaminated portions, in the population of interest. Summary statistics are also provided.

Since the model is pre-populated with data, modifications to other worksheets are possible, but not mandatory for operation. Inputs can be customized by entering three parameters to create a triangular distribution: minimum, most likely, and maximum values. While this distribution has no theoretical basis, its parameters can be intuitively defined when little data are available and the uncertainty/variability of input variables can be quickly and easily captured [12]. In addition, an individual with limited statistical experience can generally understand how the distribution's shape has been derived, making it an appropriate choice for a user-friendly model. A description of the other worksheets is provided in the Supplementary material (S1).

### Model details

A list of default input parameters and brief descriptions are reported in Table 1. Full descriptions can be found in the Supplementary Material (S2). Key aspects and calculations for the QMRA are provided below, and these calculations remain constant in the model regardless of user input values.

**Table 1.** Input parameters for the exposure assessment component

Input	Units	Source(s)	Description (Supplementary material location)	Distribution/values
Portion size	g	[19, 34]	Minimum is the lowest portion size reported by Australian dietary habit surveys. Mode and maximum are based on the portion sizes obtained from the 1994–1996 cycle of the United States’ Continuing Survey of Food Intakes by Individuals (S2.1)	Delicatessen meat: Triangular(15, 56, 113)
				Hot dogs: Triangular(42, 57, 121)
Time on retail display	days	[34]	Derived from a Pert distribution generated from the expert opinion of Australian national retail chain representatives (S2.2)	Delicatessen meat: Triangular(0·5, 10, 35)
				Hot dogs: Triangular(0·5, 7, 21)
Temperature at retail display	°C	[15]	Pre-packaged lunch meat temperature at retail establishments based on the 2007 US Cold Temperature Evaluation (S2.3)	Laplace(4·4444, 3·1351)
Time of transportation to home	h	[15]	Time elapsed between retail purchase and home refrigeration for pre-packaged lunch meats based on the 2007 US Cold Temperature Evaluation (S2.4)	Loglogistic[-0·18788, 1·3267, 5·5095, Truncate(0·2, 3·8333333)]
Temperature of transportation to home	°C	[15]	Temperatures of pre-packaged lunch meat at retail and prior to home refrigeration based on the 2007 US Cold Temperature Evaluation (S2.5)	Uniform (time of transportation to home), Weibull[3·0893, 11, shift(-1·6763)]
% of product refrigerated	%	[24, 34]	Minimum based on a web-based survey of US adults; Maximum based on two values reported in the US FDA/FSIS risk assessment (S2.6)	Delicatessen meat: 100
				Hot dogs: Uniform[55·8 (Uniform(97–91·3)]
Time stored in refrigerator	days	[16]	Based on a national representative survey of US adults (*n* = 2428). Values were estimated using the refrigeration time to first consumption and last consumption of hot dogs and delicatessen meats sliced by the distributor (S2.7)	General: discrete ({first consumption, last consumption}, {probability fully consumed at first, 1 – probability})
				Delicatessen meat: discrete ({Weibull(0·799, 3·91), Weibull(1·29, 20·5)}, {0·08, 0·92})
				Hot dogs: Discrete ({Weibull(0·779, 4·72), Weibull(1·29, 20·5)}, {0·13, 0·87})
Temperature of refrigerator	°C	[16]	Based on a national representative survey of US adults (*n* = 2037) (S2.8)	Laplace(4·06, 2·31)
Counter storage time	days	[40]	Based on experimental thermal profiles of cold smoked salmon (S2.9)	Exponential (0·3) truncated at maximum of 1
Counter-top temperature of product	°C	[13]	The refrigerator temperature is the minimum. The average room temperature in Canadian households represents the maximum (S2.10)	Uniform[Laplace(4·06, 2·31), Uniform(20, 22)]
Portions that contaminate environment	%	[20]	Prevalence of unwashed cutting boards during the food preparation of raw and heated ready-to-eat foods (S2.11)	Triangular(3, 26, 38)
Transfer rates for contamination	%	[21]	Based on transfer rates from raw chicken to cutting boards, and from cutting boards to warm and cool chicken (S2.12)	See Supplementary material, Table S1·12
% of portions consumed raw	%	[24, 34]	Based on a web-based survey of US adults and a distribution reported in the US FDA/FSIS risk assessment (S2.13)	Delicatessen meat: 100
				Hot dogs: Uniform[0·2, Triangular(4, 7, 10)]
Reheating time	min	[23]	Manufacturer recommended cooking instructions for hot dogs. Included variation in hot dog size, and heating method. (S2.14)	Delicatessen meat: n.a.
				Hot dogs: Uniform(0·5, 9)
Internal temperature of reheated hot dogs	°C	[19]	Based on estimates used in the US FDA/FSIS *L. monocytogenes* risk assessment (S2.15)	Delicatessen meat: n.a.
				Hot dogs: Triangular[54, Uniform(69–73), 77]
Referent *D, T, Z* values	s, °C	[14]	Based on thermal inactivation of *L. monocytogenes* in beef frankfurters (S2.16)	*D*_ref_: 3.2 s
				*T*_ref_: 70 °C
				*Z* value: 5·47 °C

#### Exposure assessment

##### Growth models and storage conditions

The model incorporates six sets of growth parameters specific to *L. monocytogenes* on RTE beef, ham, and turkey delicatessen meats, each with or without growth inhibitors [17]. The growth parameters for RTE beef delicatessen meats were adopted for hot dogs in the absence of other suitable data. Growth inhibitors include lactate and diacetate, both of which are permitted food additives in Canada [18]. Growth is modelled throughout retail display, transport to home, consumer refrigerated storage, and counter-top storage. Time elapsed between production and retail display is considered to determine whether the initial lag phase has been exceeded and growth has occurred (Supplementary material, S2.17). Growth rates are adjusted for storage temperatures at each stage based on the US FDA/FSIS [19] equation:
1
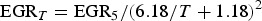


where EGR_*T*_ represents the calculated exponential growth rate (log_10_ c.f.u./g per day), EGR_5_ represents the exponential growth rate at 5 °C and *T* represents the storage temperature (°C). The maximum concentration of *L. monocytogenes* is limited to uniform ranges between 4·5–5·5, 6–7, and 7·5–8·5 log_10_ c.f.u./g for temperatures <5 °C, 5–7 °C, and >7 °C, respectively, in accordance with previous risk models [17, 19]. Freezer storage temperature is not included because frozen foods do not support growth of *L. monocytogenes* [7].

##### Cross-contamination

The likelihood of cross-contamination by the consumer is based on the prevalence of using unwashed cutting boards during preparation of raw and cooked RTE foods [20] (Supplementary material, S2.11). Where no cross-contamination occurs, the level of *L. monocytogenes* remains unchanged and is carried forward to the thermal inactivation module. For all other portions, cross-contamination is modelled in two stages, using distinct transfer rates: (1) transfer of *L. monocytogenes* from unheated meat to the preparation environment and (2) subsequent transfer from the preparation environment to either unheated or heated meat prior to consumption [21]. The latter stage reduces the overall amount of bacteria eventually consumed for unheated products, because a portion of bacteria are assumed to remain on the food preparation surface. The bacterial load transferred to the preparation surface is important to capture for reheated products such as hot dogs, as it is not subjected to inactivation during reheating, whereas the load remaining on the product can be inactivated. The estimates are defined by discrete uniform distributions based on transfer rates under several experimental conditions (Supplementary material, S2.12).

##### Thermal inactivation (reheating)

*L. monocytogenes* inactivation is modelled using the thermal inactivation equation [22]:
2
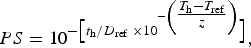


where *PS* is the probability of survival, *t*_h_ is the heating time, *T*_h_ is the heating temperature, *D*_ref_ is the reference decimal reduction time (the heating time required to kill 90% of the organisms), *T*_ref_ is the reference temperature, and *z* is the temperature required for a 1 log reduction in the *D* value. Heating time and temperature are each defined by triangular distributions. Heating times are based on the manufacturer's cooking instructions [23] and heating temperatures are based on the US FDA/FSIS risk assessment thermal inactivation module for hot dogs [19] (Supplementary material, S2.14–15). The *D*_ref_, *T*_ref_, and *z* values are specific to the thermal inactivation of *L. monocytogenes* in beef frankfurters [14] (Supplementary material, S2.16). The probability of survival is used to approximate *L. monocytogenes* concentrations (c.f.u./g) remaining in reheated portions following thermal inactivation.

The model is set so that between 90% and 99·8% of hot-dog portions are reheated prior to consumption [19, 24]. It is conservatively assumed that 100% of RTE delicatessen meats were consumed without a reheating step performed by the consumer.

##### Risk factors and comorbidity

Given that host risk factors such as pre-existing health conditions drive vulnerability to listeriosis, Canadian demographic data describing occurrence of select risk factors were collected (Supplementary material, S3) to allow for comparison of risks across subpopulations in Canada. Portions are divided in vulnerable subpopulations based on risk factor prevalence in the selected region. The risk factors include age ⩾65 years, pregnancy, and several other diseases/conditions which alter the immune system (cancer, organ transplant patient, etc.). The subpopulations used in the model are described in Table 2, and were selected for inclusion primarily because dose-response information was available. A literature search was conducted to identify prevalence estimates of risk factors across Canada and, where possible, within each province and territory. When available, national, provincial, and territorial estimates were preferentially captured from government websites and relevant health organizations. Missing data were sought through primary research articles. Where province- and territory-specific estimates were unavailable, national estimates were used.

**Table 2.** Subpopulation descriptions and their corresponding relative risk values and listeriosis dose-response model R parameters

Subpopulation	Description	Relative risk [30]	*R* parameter (log_10_ Normal distribution [10])	
			*μ*	*σ*
<65 years healthy	Population <65 years with no conditions	Reference group	−14·11	1·62
⩾65 years	Population ⩾65 years with no conditions	13·9	−12·83	1·62
Cancer (haematological)	Leukaemia, Hodgkin's lymphoma, non-Hodgkin's lymphoma, multiple myeloma	373·6	−11·02	1·62
Cancer (non-haematological)	Breast, brain, ENT, gastrointestinal, gynaecological, kidney, liver, lung, prostate cancers	54·8	−12·11	1·62
Diabetes	Type I, Type II	7·6	−13·13	1·62
Dialysis/liver disease	Dialysis: haemodialysis, peritoneal dialysis; liver disease: hepatitis A, B, C	149·4	−11·56	1·62
Heart disease	Self-reported heart disease	5·4	−13·30	1·62
HIV/AIDS	HIV, or HIV/AIDS	47·4	−12·19	1·62
Inflammatory disease	Rheumatoid arthritis, Crohn's disease, colitis	58·5	−12·08	1·62
Organ transplant	Heart, intestinal, kidney, liver, lung, and pancreas transplant patients	163·7	−11·51	1·62
Pregnancy	Total number of live births + fetal loss + abortions/population * 0·75	116	−11·70	1·62

ENT, Ear, nose and throat.

Previous listeriosis risk assessments estimated the proportion of susceptible individuals by summing prevalence estimates across risk factors [11, 25, 26]. However, this does not account for comorbidities, or occurrence of more than one risk factor for listeriosis in any given individual, and may lead to double counting. This model considers ten risk factors (in addition to the general population aged <65 years with no conditions). Disregarding the potential for multiple risk factors for listeriosis would lead to an overestimation of the vulnerable population (e.g. summing prevalence of individual risk factors without accounting for comorbidity results in approximately 40% of the population considered as vulnerable). Unfortunately, there is a paucity of data on the occurrence of multiple risk factors specific to listeriosis. As an approximation, Canadian comorbidity data available for a suite of health conditions were used to estimate the proportion of the Canadian population with either zero risk factors or at least one risk factor for listeriosis [27]. Using these data combined with age distributions from Statistics Canada [28], the modeled population was divided into five primary categories; <65 years with no conditions, <65 years with ⩾1 condition, ⩾65 years with no conditions, ⩾65 years with ⩾1 condition, and pregnant women. The latter four categories are all considered vulnerable to listeriosis. Population segments with ⩾1 condition were further divided by their primary condition as determined by the relative prevalence of risk factors in the chosen location. It was assumed that pregnant women do not have additional risk factors. Portions are divided in the subpopulations according to their prevalence as described above. Sex-specific population and prevalence estimates were used only in intermediate calculations to account for key variation in some risk factors (e.g. prostate cancer, pregnancy). Due to the absence of sex-specific data on all risk factors and sex-specific listeriosis risks, outputs are pooled for both sexes.

#### Hazard characterization

Exponential dose-response models of the general form below are used:
3
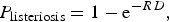

where *P*_listeriosis_ is the probability of listeriosis, *D* is the dose consumed, and *R* is the fitted parameter specific to each subpopulation of interest. The general model was obtained from Ross *et al.* [11], which was based on an exponential model [29]. Eleven dose-response models are used, each with previously derived subpopulation-specific distributions of *R* parameters (Table 2) [10]. The *R* parameters capture variability in subpopulations, within subpopulations, and in *L. monocytogenes* strains [10]. The subpopulation-specific *R* parameters were derived from listeriosis relative risk estimates identified in a French population by Goulet *et al.* [30] (Table 2).

#### Risk characterization

Integration of the exposure assessment and hazard characterization components yields the probability of listeriosis for each subpopulation/portion category combination, which is then multiplied by the number of portions consumed by each subpopulation to predict the number of listeriosis cases for each subpopulation. Due to the stochastic nature of the model and lack of truncation bounds on the distributions of *R* values used in the dose-response analyses, in some model iterations the *R* value for a given risk factor is less than the value for the same age group with no conditions. In these cases, the greater *R* value was implemented in the dose-response model for the vulnerable subpopulation. Although it is acknowledged that the dose-response functions account for strain variability, this adjustment was made as a conservative measure. Estimated cases are summed across all subpopulations to provide an estimate of risk for the entire population.

#### Simulations

Demonstrative sets of simulations were performed to compare products, growth inhibitor use, regions, and subpopulations, using hypothetical user inputs. Although it was preferred to simulate a historical contamination event, adequate data to sufficiently replicate a previous outbreak, such as the 2008 outbreak in Canada [6], were unavailable. For all simulations, 100 000 iterations were run with a fixed initial seed. Triangular distributions (minimum, most likely, maximum) were used to characterize the user inputs: number of potentially contaminated portions (80000, 100000, 120000), contamination prevalence (50%, 60%, 65%), and the *L. monocytogenes* contamination level at retail (0·04, 2·34, 254 c.f.u./g). Prevalence of contamination approximated conditions measured from previous *L. monocytogenes* outbreaks in RTE foods [31–33]. The 5th, 50th, and 95th percentiles of the cumulative distribution of levels of *L. monocytogenes* in Australian processed meats in Ross *et al.* [34] were adopted. Spearman's rank-order correlation coefficients, as well as summary statistics such as median, 5th, and 95th percentiles of outputs, were derived from @Risk outputs.

## RESULTS

### Product and growth inhibitor comparison

**Fig. 2.**
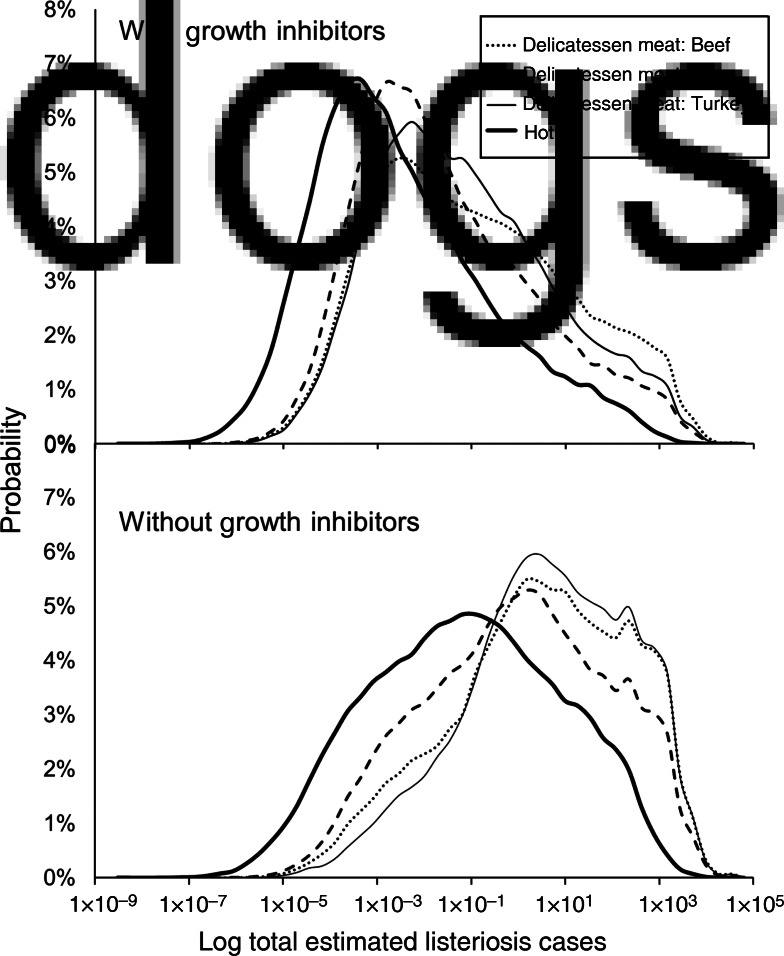
Probability distributions of total estimated listeriosis cases for consumption of contaminated beef, ham, and turkey delicatessen meats, and hot dogs, with and without growth inhibitors in Canada. Distributions result from Monte Carlo simulations using Latin Hypercube Sampling with 100 000 iterations. Distributions are shown in a log scale, and central tendencies are not constant across log transformations. (See Table 3 for median estimates of listeriosis cases.)

Hypothetical contamination events were simulated to compare products and growth inhibitor use, assuming equal product distribution across Canada. The medians, 5th, and 95th percentiles of listeriosis cases predicted are presented in Table 3. The model predicted approximately two orders of magnitude fewer listeriosis cases for products with growth inhibitors compared to products without growth inhibitors. The median number of estimated listeriosis cases ranged from 0·0022 to 0·089 and 0·11 to 6·6 cases for products with and without growth inhibitors, respectively. Distributions of estimated listeriosis cases for each product are depicted on a logarithmic scale in Figure 2. This figure provides an indication of the relative differences in products, but note that the central tendencies are not constant across log transformations. While similar patterns in products are apparent, the model predicts a wide range of estimated listeriosis cases for each product scenario.

**Table 3.** Median listeriosis cases and the 5th and 95th percentile estimates for each of the products following a simulated *L. monocytogenes* contamination event in Canada, as determined by Monte Carlo simulations using Latin Hypercube Sampling with 100 000 iterations. Relative differences as compared to the product with the greatest public health risk are provided to demonstrate the utility of the model for comparing various product and product treatment scenarios

Product	Median listeriosis cases (5th percentile, 95th percentile)		Median cases relative to turkey without growth inhibitors (%)
Beef delicatessen meat with growth inhibitors	8·9 × 10^−2^	(1·8 × 10^−4^, 7·3 × 10^2^)	1·3
Ham delicatessen meat with growth inhibitors	1·9 × 10^−2^	(1·2 × 10^−4^, 2·0 × 10^2^)	0·28
Turkey delicatessen meat with growth inhibitors	6·0 × 10^−2^	(2·0 × 10^−4^, 3·5 × 10^2^)	0·91
Hot dogs with growth inhibitors	2·2 × 10^−3^	(1·2 × 10^−5^,2·0 × 10^1^)	0·03
Beef delicatessen meat without growth inhibitors	5·0	(1·3 × 10^−3^, 2·1 × 10^3^)	75
Ham delicatessen meat without growth inhibitors	1·5	(6·1 × 10^−4^, 1·5 × 10^3^)	22
Turkey delicatessen meat without growth inhibitors	6·6	(3·5 × 10^−3^, 2·1 × 10^3^)	Reference
Hot dogs without growth inhibitors	1·1 × 10^−1^	(5·5 × 10^−5^, 2·1 × 10^2^)	1·6

The impact of model input variables on the number of cases was assessed through comparison of Spearman's rank order correlation coefficients. Input variables with the greatest coefficients for each scenario ranged from 0·30 to 0·43, were product dependent, and included consumer refrigerator storage temperature, consumer refrigerated storage time, exponential growth rate, retail storage temperature, and storage temperature prior to retail display. The latter was used to determine lag time for *L. monocytogenes* growth, and correspondingly, lag time was negatively correlated with public health impacts (−0·13 to −0·27).

**Fig. 3.**
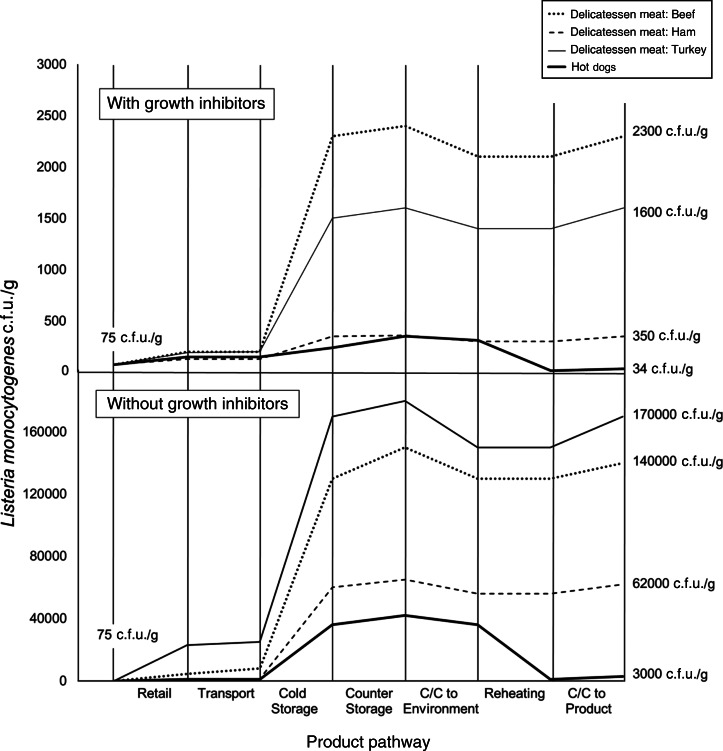
*Listeria monocytogenes* median contamination levels throughout the retail-to-consumption pathway for beef, ham, and turkey delicatessen meats, and hot dogs with and without growth inhibitors. Contamination levels are based on weighted average medians across all portion types taken at the beginning and end of each step in the pathway, as determined using Monte Carlo simulations using Latin Hypercube Sampling with 100 000 iterations. C/C, Cross-contamination. Initial concentrations were equivalent across products, and final median concentrations are indicated. Each vertical line indicates the beginning of one stage and the end of the previous stage. Contamination levels at entry and exit of each stage are linked with straight lines for ease of visualization only, and do not indicate linear increases within stages.

Median *L. monocytogenes* contamination levels (c.f.u./g) were greater at all exposure points prior to consumption compared to initial levels at retail for all delicatessen meats (Fig. 3). Contamination levels on products increased from retail through counter-top storage, but not following cross-contamination and reheating. The greatest relative increase in *L. monocytogenes* levels occurred during cold home storage. The largest decrease in contamination levels occurred during reheating of hot dogs. The ranking of final contamination level in products corresponded with the median number of listeriosis cases (Table 3, Fig.3).

### Risk factor comparison

**Fig. 4.**
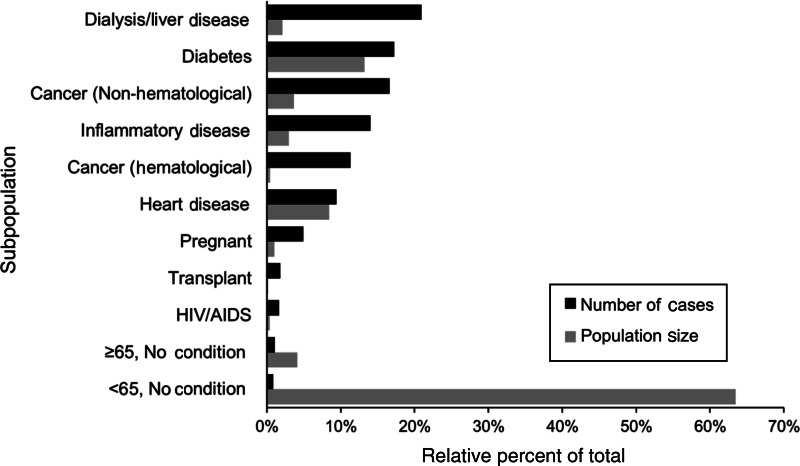
Relative percent of listeriosis cases in eleven subpopulations given consumption of contaminated turkey delicatessen meat with growth inhibitors in Canada, as determined using Monte Carlo simulation using Latin Hypercube Sampling with 100 000 iterations. Relative size of each subpopulation in Canada is also shown. Relative population size is equivalent to the relative number of contaminated portions consumed by each subpopulation. (See Table 4 for median estimates of listeriosis cases and portions.)

Simulation results for risks from turkey delicatessen meat with growth inhibitors in Canada were further studied to evaluate the distribution of model results by subpopulation. The median number of portions consumed, median probability of illness averaged across portion types, and median number of listeriosis cases, along with the 5th and 95th percentiles, for each subpopulation are presented in Table 4. The highest probability of illness was predicted in the haematological cancer risk factor subpopulation, followed by the transplant, dialysis/liver disease, and pregnant risk factor subpopulations. The relative proportion of contaminated product consumed by each subpopulation is compared with their respective number of simulated cases of listeriosis in Figure 4. Portions consumed directly reflect the relative prevalence of the risk factors in the population, as described previously. The largest number of listeriosis cases was predicted in the dialysis/liver disease risk factor subpopulation followed by the diabetes, non-haematological cancer, inflammatory disease, and haematological cancer risk factor subpopulations. Subpopulations with no risk factor (<65 years with no condition) accounted for only 1% of all listeriosis cases, despite consuming 63% of the simulated portions. A marginally greater number of cases were predicted in subpopulations aged ⩾65 years with no other risk factors, despite the relatively low prevalence of these individuals in Canada (approximately three quarters of individuals aged ⩾65 years across Canada were considered to have at least one other known risk factor in the model, and are therefore represented in the other risk-factor classifications). Individuals undergoing dialysis and/or with liver disease accounted for 21% of all listeriosis cases, but consumed only 2% of the contaminated portions from the simulated event.

**Table 4.** Median portions consumed, probability of illness, listeriosis cases and respective 5th and 95th percentile estimates for the consumption of contaminated turkey delicatessen meats with growth inhibitors in each subpopulation included in the model, assuming a hypothetical contamination event. Probability of illness was determined as a weighted average across all portion types. Results were determined by Monte Carlo simulations using Latin Hypercube Sampling with 100 000 iterations

Subpopulation	Median consumed portions (5th percentile, 95th percentile)		Median probability of illness (5th percentile, 95th percentile)		Median listeriosis cases (5th percentile, 95th percentile)	
<65 years, no condition	3·7 × 10^4^	(3·1 × 10^4^, 4·3 × 10^4^)	1·2 × 10^−9^	(3·6 × 10^−13^, 2·3 × 10^−5^)	4·3 × 10^−5^	(1·3 × 10^−8^, 8·7 × 10^−1^)
⩾65 years, no condition	2·4 × 10^3^	(2·0 × 10^3^, 2·8 × 10^3^)	2·2 × 10^−8^	(6·8 × 10^−12^, 4·5 × 10^−4^)	5·3 × 10^−5^	(1·6 × 10^−8^, 1·1 × 10^0^)
Cancer (haematological)	2·4 × 10^2^	(2·0 × 10^2^, 2·8 × 10^2^)	1·4 × 10^−6^	(4·7 × 10^−10^, 2·9 × 10^−2^)	5·7 × 10^−4^	(4·3 × 10^−7^, 8·7 × 10^0^)
Cancer (non-haematological)	2·2 × 10^3^	(1·8 × 10^3^, 2·5 × 10^3^)	1·1 × 10^−7^	(3·7 × 10^−11^, 2·4 × 10^−3^)	8·3 × 10^−4^	(8·9 × 10^−7^, 1·1 × 10^1^)
Diabetes	7·7 × 10^3^	(6·5 × 10^3^, 9·0 × 10^3^)	1·1 × 10^−8^	(3·5 × 10^−12^, 2·3 × 10^−4^)	8·7 × 10^−4^	(1·1 × 10^−6^, 1·0 × 10^1^)
Dialysis/liver disease	1·2 × 10^3^	(9·8 × 10^2^, 1·5 × 10^2^)	4·1 × 10^−7^	(1·3 × 10^−10^, 8·1 × 10^−3^)	1·1 × 10^−3^	(9·3 × 10^−7^, 1·5 × 10^1^)
Heart disease	4·9 × 10^3^	(4·1 × 10^3^, 5·7 × 10^3^)	7·3 × 10^−9^	(2·4 × 10^−12^, 1·7 × 10^−4^)	4·7 × 10^−4^	(5·9 × 10^−7^, 5·6 × 10^0^)
HIV/AIDS	2·4 × 10^2^	(2·0 × 10^2^, 2·9 × 10^2^)	9·6 × 10^−8^	(2·9 × 10^−11^, 2·0 × 10^−3^)	8·3 × 10^−5^	(8·7 × 10^−8^, 1·1 × 10^0^)
Inflammatory disease	1·7 × 10^3^	(1·5 × 10^3^, 2·0 × 10^3^)	1·3 × 10^−7^	(3·9 × 10^−11^, 2·5 × 10^−3^)	7·0 × 10^−4^	(7·3 × 10^−7^, 9·0 × 10^0^)
Organ transplant	9·5 × 10^1^	(8·0 × 10^1^, 1·1 × 10^2^)	4·5 × 10^−7^	(1·5 × 10^−10^, 9·5 × 10^−3^)	9·3 × 10^−5^	(8·5 × 10^−8^, 1·4 × 10^0^)
Pregnant	6·0 × 10^2^	(5·1 × 10^2^, 7·0 × 10^2^)	3·0 × 10^−7^	(8·8 × 10^−11^, 5·8 × 10^−3^)	2·5 × 10^−4^	(1·3 × 10^−7^, 4·1 × 10^0^)

Two additional contamination event scenarios were tested using the model to compare differences in risks in subpopulations aged <65 years and ⩾65 years, such as what might happen if product is generally distributed to grocery stores, etc., or distributed solely to institutions comprised mostly of at-risk subpopulations such as long-term-care facilities. For these simulations, beef delicatessen meat with growth inhibitors was assumed to be distributed either generally across the province of Ontario, or solely to subpopulations aged ⩾65 years. The median number of resulting listeriosis cases in the general population and the ⩾65 years population was 9·1 × 10^−2^ (5th percentile: 1·9 × 10^−4^, 95th percentile: 7·1 × 10^2^) and 1·7 × 10^−1^ (5th percentile: 3·1 × 10^−4^, 95th percentile 1·4 × 10^3^), respectively. Therefore, the number of estimated listeriosis cases was 1·9 times greater in the population aged ⩾65 years compared to the general population.

## DISCUSSION

Absolute risk values should be interpreted with caution for any given simulation due to uncertainty in model inputs. Rather, the purpose of this model is to compare risks under different scenarios, or for different products, interventions, regions, or risk factors. The model can be used to explore conditions and outcomes under different circumstances, such as simulating a contamination event and/or outbreak. The discussion below is provided in reference to the simulated hypothetical contamination events; while the intention of this paper is primarily to introduce the user-friendly model, a focus on the simulated results is prudent to provide an indication of how model results can be interpreted and used to inform public health activities.

Previous risk assessments have demonstrated that *L. monocytogenes* growth on RTE meats and resulting listeriosis cases are dependent on the product and its additives [11, 17, 35]. Thus, it was important to include the ability to evaluate listeriosis risk in different RTE meats and growth inhibitor uses in the model. Consumption of turkey delicatessen meat without growth inhibitors resulted in the highest number of listeriosis cases in the hypothetical scenario (Table 2). As expected, presence of growth inhibitors led to fewer listeriosis cases for all RTE meats considered in this study; however, the relative reduction in cases due to growth inhibitor use differed by RTE meat type and initial contamination level. Use of growth inhibitors led to a 110-fold reduction in the median number of listeriosis cases for turkey delicatessen meat, followed by ham delicatessen meat, beef delicatessen meat, and hot dogs, with 78-fold, 56-fold, and 49-fold reductions, respectively, using the simulation inputs previously described. However, arbitrarily lowering the initial contamination levels to reflect a triangular (0·04, 1, 2 c.f.u./g) distribution resulted in corresponding 952-, 279-, 381-, and 116-fold reductions. Use of growth inhibitors had a greater relative impact on risks from beef compared to ham delicatessen meat at this lower contamination level scenario. Such differences highlight the importance of using event-specific inputs where possible, as general ‘rules of thumb’ might not apply across all possible contamination scenarios. The effects of growth inhibitors are dependent on several properties (e.g. temperature, pH, fat content), which may explain variation in products [36–38].

Within each category of growth inhibitor use, hot dogs caused fewer listeriosis cases than delicatessen meats (Table 2). This is consistent with Ross *et al.* [11] and is attributed to differences in consumer storage and reheating practices. Of delicatessen meats, fewer cases were consistently estimated for ham. Median lag times for ham exceeded those for beef and turkey. Such extended lag times resulted in shorter growth periods and fewer predicted listeriosis cases. While the growth rates and lag times incorporated in this model were distributions based on several studies [17], recent studies on *L. monocytogenes* growth [35, 39] could be incorporated into the distributions based on user needs.

The importance of *L. monocytogenes* growth during storage is noted in several risk assessments [19, 40–42], and not surprisingly echoed in our results. The majority of *L. monocytogenes* growth occurred during consumer cold storage and although growth during retail storage was not as pronounced, correlation with model outputs remained high, especially in products without growth inhibitors.

Regional comparisons provide a framework for integration of demographic, epidemiological, and dose-response data to compare risks across regions for decision making purposes. Age demographics and the prevalence of listeriosis risk factors varied in the regions considered in the model, and therefore regional distribution of potentially contaminated product is important. However, regional prevalence data, although available (see Supplementary material, S3), were not derived using identical approaches in all cases. These data are incorporated in the model, but a summary of regional comparisons is not presented herein given differences in data derivation for the provinces and territories.

Generally, previous listeriosis risk assessments have summed national risk factor prevalence and used adjusted dose-response models to account for two subpopulations; the healthy and the vulnerable [11, 19]. This model builds on this approach by incorporating region-specific demographic data for several risk factors, accounting (albeit imperfectly) for comorbidities, and providing estimates of risk for ten vulnerable subpopulations alongside the general population. The model is amenable to modifications to allow for greater regional specificity in model components (e.g. portion sizes), and smaller-scale regional assessments (e.g. census subdivisions, municipalities, institutions, etc.). At present, identically derived provincial and territorial demographic data are lacking, and data are unavailable to tailor the model to an even smaller spatial scale. However, in the event of a suspected contamination event or outbreak, health authorities may be able to obtain product distribution and demographic information to allow for such modifications. The regional selection in the model allows for comparison of inter- or intra-regional risks for estimating listeriosis cases and targeting interventions such as public health messaging to vulnerable groups.

Individual-level risk and population-level risk of listeriosis differ in their computation and utility. Individual-level risk for example, is often represented by relative risk in epidemiology studies or probability of illness in QMRAs [19, 34, 43]. The relative estimated probabilities of illness across vulnerable subpopulations included in this model align with relative risk values recently derived and used in the model [10]. Individuals with haematological cancer had the highest probability of listeriosis per portion, followed by transplant patients and individuals on dialysis/with liver disease (Table 4). These subpopulations represent high-risk individuals, and would benefit most from interventions at the primary care level (e.g. physician advice to at-risk patients to heat RTE foods to temperatures lethal to *L. monocytogenes*, or avoid specific higher-risk foods such as RTE delicatessen meats).

The model represents population-level risk as the number of estimated listeriosis cases. This estimate accounts for both individual-level risk, and risk factor prevalence in the population. Subpopulations with at least one risk factor accounted for 99% of all predicted listeriosis cases, which is consistent with epidemiological studies where 98% of individuals with listeriosis had at least one pre-existing condition [44]. The Canadian subpopulation on dialysis or with liver disease was associated with the most listeriosis cases in the model. From a risk assessment perspective, in outbreak situations and exploratory modelling, population-level risk is important as it identifies the populations where one might expect the most cases to occur. The subpopulation comparison indicated that 80% of cases are expected to occur in 20% of the population: those with liver disease and/or undergoing dialysis, diabetes, cancer, and inflammatory disease. By targeting interventions such as public health messaging at these specific risk factor groups, significant listeriosis burden could be reduced using a fraction of the resources required to target the entire population.

The model predicted that distribution of a contaminated batch of RTE meat to a population aged exclusively ⩾65 years (e.g. a long-term-care facility) led to nearly double the number of cases compared to distribution to a population comprising all ages. This reiterates the importance of both age, and risk factors that accumulate with age, in listeriosis outbreaks [30, 45–47]. Individuals aged ⩾65 years are more likely to have at least one additional chronic, often immunosuppressive, condition [27], as was the case in the 2008 listeriosis outbreak [6]. This tool, had it existed at that time, could have been used to provide insights on risks posed to vulnerable populations to which different RTE meat products were distributed (including long-term and acute-care facilities), and support preventative strategies.

There are several key limitations to the model and its inputs. Some inputs to the model were not well-characterized in the literature or publicly accessible databases. Region-specific risk factor prevalence values were, in some cases, unavailable. In these cases, national prevalence data were used as a surrogate input. It is expected that regional variations in risk factor prevalence exist, and should be implemented in the model when data are available. For some inputs, specifically demographic data for cancer, comparable data across regions were not available (e.g. 10-year *vs*. lifetime prevalence values). Additionally, the risk factors and relative risk estimates included in the model were originally derived for France [30], and could lack generalizability. Since the relative risk factors were derived based on observed cases of listeriosis, they inherently account for differences in general consumption patterns in vulnerable subpopulations. However, consumption patterns of RTE meats in at-risk groups could differ in Canada. Relative risk values for listeriosis should be parameterized using Canadian data and implemented in risk models.

While the methodology used to account for comorbidity improves, in our opinion, upon previous *L. monocytogenes* QMRA models, it is based on comorbidity information for a small subset of conditions [27]: arthritis, cancer, chronic obstructive pulmonary disease, diabetes, heart disease, high blood pressure, and mood disorders. Only some of these conditions were identified as risk factors for listeriosis, and other risk factors were not included in comorbidity estimates. Nevertheless, using these data provided a generic indication of occurrence of comorbidities in the Canadian population, which is preferable to simply summing the prevalence of all conditions (which will overestimate the total vulnerable population as approximately 40%) and ignoring co-occurrence of risk factors to listeriosis altogether. Estimates of the total vulnerable population calculated in the model ranged from 20–39%, depending on region considered. The World Health Organization (WHO) and Food and Agriculture Organization (FAO) [43] estimated that 22·4% of the Canadian population was susceptible to invasive listeriosis. However, age demographic data used in that study are over a decade old, and the proportion of Canadians aged ⩾65 years was 12·5%, compared to 15·7% used in this model. In addition, the prevalence of other risk factors across all age groups was 31·4% herein, compared to 4·6% used (pregnancy and immunodeficiency) in the WHO/FAO study [43].

Several risk factors, including but not limited to alcoholism, antacid use, corticosteroid therapy, and laxative use were identified, but dose-response data were unavailable and therefore they were not considered in the model. Inclusion of these risk factors could have a profound impact on the model results. For example, when prevalence data for alcoholism are included in the model (estimated as the prevalence of chronic drinking in Canada as defined by Health Canada [48], or 14·4% of the population aged ⩾15 years), and arbitrarily adopting the dose-response *R* value for liver disease, the projected number of cases in a simulated contamination event increased by 21%. The model does not explicitly incorporate additive or synergistic risk in those with multiple risk factors, although the methods in the derivation of relative risk values may partially account for this. Goulet *et al.* [30] classified patients with >1 underlying risk factor according to the most immunosuppressive condition. Further research on listeriosis risk factors, their overlap, and their resulting contributions to the risk of listeriosis would greatly benefit subpopulation risk characterization.

This model was designed to be user friendly; however, in doing so, some aspects of more complex or all-encompassing QMRA models were not included. While the focus herein has been on pre-packaged delicatessen meat, delicatessen meat sliced at retail should also be considered. Although it is unlikely these products would be distributed to hospitals or long-term-care homes, the risk associated with counter-sliced meats is considerably greater than prepackaged meats [49, 50], thus possibly representing an important outbreak source for the general population. Additionally, consumption habits in vulnerable subpopulations should be included when data are available, as they are likely to differ [51, 52].

The QMRA model provides several novel aspects. It provides a simple user interface to allow for use by public health officials not necessarily well-versed in modelling, similar to other tools [9]. However, unlike these generic tools, it incorporates uncertainty and variability in inputs, and is pre-populated for a specific pathogen and suite of food products. Therefore, it is an easily modifiable tool that can be adapted to reflect the particular circumstances of *L. monocytogenes* contamination events in Canada. Further, the integration of several regions, products, and vulnerable subpopulations allow for risk comparisons and the identification of high priority populations to direct public health interventions. Example simulations presented herein provide an indication of how the tool can be used to identify higher risk food-treatment combinations and distribution scenarios.

## Supplementary material

For supplementary material accompanying this paper visit https://doi.org/10.1017/S0950268816000327. click here to view supplementary material
