# Supplementary material for: Comparing listeriosis risks in at-risk populations using a user-friendly quantitative microbial risk assessment tool and epidemiological data
Source: Epidemiol Infect. 2016 Mar 28;144(13):2743–58. doi: 10.1017/S0950268816000327 (PMC9150414; doi:10.1017/S0950268816000327)
Supplement: Supplementary file 1 [file S0950268816000327sup001.docx]

# Supplementary Material

### S1. Basic Description of Model and Worksheets

The model is implemented in @Risk^TM^ 6.3, an add-in for Microsoft® Office Excel which employs Monte Carlo analysis with Latin Hypercube Sampling throughout the simulation process. The model is divided into six worksheets; *Instructions, Model Input and Summary, Exposure Assessment, Hazard/Risk Characterisation, Risk Factors,* and *Supplementary Parameters*. Users can operate the model through the *Model Input and Summary* sheet, where basic information is entered and summary results are displayed. The user must provide, at a minimum, the type of food product (RTE beef, ham or turkey deli meat, or hot dogs, with or without growth inhibitors), the number of servings potentially contaminated (e.g. the number of servings produced in one batch or bracketed batches at a processing facility), the prevalence and initial contamination level of the product at retail and the geographic location (Canada-wide, or by province or territory) where the food is distributed and consumed. Once the user has entered the minimum required data, the simulation can be run and results displayed. The model presents the calculated risk as a probability density function for the total number of cases predicted to occur from the potentially contaminated servings, among the entire population of interest. Summary statistics are also provided in this worksheet.

If desired, model default variables can be modified using the *Exposure Assessment* sheet. The model is stochastic in nature, and implements various probability distributions to define default input parameters. However, the model is customizable so that alternate distributions or data can be entered dependent on the specific scenario and user expertise. Inputs can be customized by entering three parameters to create a triangular distribution: minimum, most likely and maximum values.

The *Susceptibility* sheet includes risk factor prevalence data and comorbidity calculations, and can be modified if more recent data are identified. The *Supplementary Parameters* sheet includes calculations required for the default inputs of the exposure assessment (e.g. growth models). Risks are calculated within the *Hazard/Risk Characterisation* sheet.

In summary, the user only needs to input information into the *Model Input and Summary* worksheet. If desired, the *Exposure Assessment* worksheet can be modified with new information relevant to the potential contamination event. The remaining worksheets contain additional information that is essential to model calculations, but generally do not require modification unless further dose-response and risk factor prevalence data become available. The model can be obtained from the corresponding author.

### S2. Exposure Assessment

#### S2.1 Portion Size (1. What is the weight of one portion?)

#### Input Values:

Deli Meat: Triangular (15, 56, 113)

Hot Dogs: Triangular (42, 57, 121)

Units: grams

Source(s): [1, 2]

Description:

The default portion sizes of deli meat and hot dogs were represented by triangular distributions. Although a survey identified portion sizes for a small Inuit Canadian subpopulation [3], a lack of generalizability led to the use of alternative sources for the distributions’ parameters. In the absence of sufficient Canadian data, it was assumed that Canadian portion sizes would be comparable to American portion sizes. The default mode and maximum portion sizes (g) were taken as the 50^th^ and 95^th^ percentiles of distributions representing the United States’ 1994-1996 Continuing Survey of Food Intakes by Individuals results (N=30,818) for deli meat and frankfurters as reported in the U.S. FDA/FSIS risk assessment [2]. The risk assessment did not report a percentile that could be used as a surrogate for the minimum value. As a result, the smallest portion sizes from a summary of Australian dietary survey results (N=28,199) as presented in the Ross *et al.* risk assessment were used as the distribution minima [1]. It is assumed that Canadian consumers eat portion sizes relatively similar to American consumers, and do not eat portions smaller than the minimum portion size of Australian consumers. Furthermore, it is assumed that average portion sizes are similar for all deli meat types. The distribution does not account for variation in portion sizes among at-risk subpopulations. Although data from the Ross *et al.* report suggest portion size could vary with age, the data were inconclusive and thus were not incorporated into the model [1].

#### S2.2 Time on Retail Display (2. How long is the product on retail display, prior to purchase?)

Input Values:

Deli Meat: Triangular (0.5, 10, 35)

Hot Dogs: Triangular (0.5, 7, 21)

Units: days

Source(s):[1]

Description:

Triangular distributions were used to represent the default storage time at retail prior to purchase (days) for deli meat and hot dogs. The distributions were populated with the minimum, mode, and maximum values reported for a Pert distribution in the Ross *et al*. risk assessment [1]. These values were based on the expert opinion of Australian national retail chain representatives. Australian data were used in the absence of Canadian retail data and it is assumed that product retail storage time would not considerably differ. Values reported for sausages (including precooked sausages) were used for hot dogs and values reported for lunch meats were used for deli meat. Alternate sources did not provide estimates specific to both lunch meats and hot dogs. The Ross *et al*. assessment contained estimates for hot dogs and deli meat but did not differentiate between pre-packaged and over-the-counter lunch meat [1]. This model focuses on the former, and due to differences in preparation, processing, and packaging, pre-packaged deli meat would likely have a longer shelf life and retail storage time. Lastly, as described in the U.S. FDA/FSIS risk assessment[2], “high storage temperatures and long storage times would not be likely to occur because this combination would lead to obvious spoilage and the food would not be consumed”; according to this reasoning, it is often inappropriate to model storage temperature and time as independent variables. Therefore retail storage times and temperatures were negatively correlated (r = -0.16). The correlation coefficient was obtained from an *L. monocytogenes* exposure assessment for cold smoked salmon [4].

*S2.3 Temperature at Retail Display (3. What is the temperature during retail display?)*

Input Values:

Deli Meat & Hot Dogs: Laplace (4.4444, 3.1351)

Units: °C

Source(s): [5]

Description:

Raw data describing pre-packaged lunch meat temperatures at various retail establishments across the United States were used to generate the distribution used in the model. The data were obtained from the EcoSure 2007 U.S. Cold Temperature Evaluation [5], in which consumers recorded the temperatures of deli meats and time information at various points along the retail to consumption pathway. Using @Risk, the raw data (N=920) were fit to a distribution and then converted from °F to °C^[[1]](#footnote-1)^. The best fit distribution was used in the model (Anderson Darling T-Statistic = 9.59). As this survey did not collect data for hot dogs, it was assumed that the values collected for pre-packaged lunch meat would be appropriate for all categories of RTE meat products considered in the model. Although no Canadian sources were available, the Canadian Food Inspection Agency recommends to store refrigerated food at 4°C[6], which falls in the approximate centre of the generated distribution. Finally, as described in the U.S. FDA/FSIS 2003 risk assessment[2], “high storage temperatures and long storage times would not be likely to occur because this combination would lead to obvious spoilage and the food would not be consumed”; according to this reasoning, it is often inappropriate to model storage temperature and time as independent variables. Therefore retail storage times and temperatures were negatively correlated (r = -0.16). The correlation coefficient was obtained from an *L. monocytogenes* exposure assessment for cold smoked salmon [4].

S2.4 Time of Transportation to Home (4. How long is the transport period from retail to home?)

Input Values:

Hot Dogs & Deli Meat: Loglogistic (-0.18788, 1.3267, 5.5095, Truncate(0.2,3.83))

Units: Hours

Source(s):[5]

Description:

Raw data on the time elapsed between retail purchase and home refrigeration for pre-packaged lunch meats were used to derive the distribution used in the model. The data were obtained from the 2007 U.S. Cold Temperature Evaluation [5], in which consumers recorded the temperatures of deli meats and time information at various points along the retail to consumption pathway. Time elapsed was calculated by subtracting the time the meat was obtained at retail from the time the when the meat was placed in the home refrigerator. Using @Risk, the raw data (N=914) were fit to a distribution and then converted to hours. The best fit distribution (Anderson Darling T-Statistic = 0.60) was truncated at the minimum and maximum elapsed times and used in the model. As this survey did not collect data for hot dogs, it was assumed that the values collected for pre-packaged lunch meat would be appropriate for all categories of RTE meat products considered in the model. Although no Canadian sources on consumer transport behaviour were available, it is assumed that American consumer behaviour is relatively similar.

#### S2.5 Temperature during Transportation to Home (5. What is the temperature during transport from retail to home?)

Input Values:

Deli Meat & Hot Dogs: Uniform(Temperature at Retail, Weibull(3.0893,11, Shift(-1.6763))

Units: °C

Source(s):[5]

Description:

Raw data on pre-packaged lunch meat temperatures at retail and prior to home refrigeration/storage were used to estimate the RTE meat temperatures during transportation. Data were obtained from the 2007 U.S. Cold Temperature Evaluation [5], in which consumers recorded the temperatures of deli meats and time information at various points along the retail to consumption pathway. The temperature of RTE meats during transportation is expected to vary between the product temperature at retail and the product temperature just prior to home refrigeration. This process was simplified for the model by using the two temperature estimates to generate the uniform distribution. The product temperature at retail was generated as described previously. The uniform distribution maxima, the product temperature just prior to home refrigeration, was similarly generated in @Risk by fitting raw data (N=919) to a distribution and converting °F to °C^[[2]](#footnote-2)^. The best fit distribution (Anderson Darling T-Statistic = 3.22) was used in the model. It was assumed that the temperature of a product will increase from retail to home and that a high retail temperature will lead to a high temperature prior to refrigeration; therefore the two estimates were positively correlated (r = 1) for the transportation section of the model. As this survey did not collect data for hot dogs, it was assumed that the values collected for pre-packaged lunch meat would be appropriate for all categories of RTE meat products considered in the model.

#### S2.6 % of Product Refrigerated (6. What percentage of portions are stored in the refrigerator or freezer?)

Input Values:

Deli Meat: 100

Hot Dogs: Uniform (55.8 - (Uniform (91.3 - 97))

Units: %

Source(s): [2, 8]

Description:

The model divides the contaminated hot dog portions into two categories of cold storage: refrigerator and freezer. The default values for the percentage of hot dog portions stored in the refrigerator was defined by a uniform distribution. Data were obtained from two sources. The minimum value for the uniform distribution was obtained from the results of a web-based survey of U.S. adults (N=1,212) [8]. The survey reported that 44.2% of households stored unopened packages of frankfurters in the freezer, therefore the remaining 55.8% were assumed to be stored in the refrigerator. The maximum value for the distribution was obtained from the U.S. FDA/FSIS 2003 risk assessment [2]. Based on the results of the AMI survey and FDA Food Safety survey in the U.S., this assessment used a uniform distribution from 3.0 to 8.7% to describe the percentage of frankfurters stored in the freezer (FDA/FSIS 2003). The model therefore uses a uniform distribution from 91.3 to 97% to define the maximum percentage of hot dogs stored in the refrigerator. Since freezing deli meat is not common practice, 100% of the contaminated deli meat portions are assumed to be stored in the refrigerator. Although no Canadian sources were available, it was assumed that American consumer behaviours are similar to those of Canadian consumers.

*S2.7 Time Stored in Refrigerator (7. How long is the product stored in the refrigerator?*

Input Values:

General: Discrete ({first consumption, last consumption}, {probability fully consumed at first, 1-probability fully consumed at first})

Hot dogs: Discrete ({Weibull (0.779,4.72), Weibull (1.12, 12.5)}, {13%, 87%})

Deli meat: Discrete ({Weibull (0.799,3.91), Weibull (1.29, 20.5)}, {8%, 92%})

Units: days

Source(s):[7]

Description:

A study by Pouillot *et al*.[7], which used data from a national representative survey of U.S. adults (N=1,192), was used to estimate the amount of time RTE products are stored in the refrigerator. RTE products are not always wholly consumed on the first occasion they are eaten, and thus distributions were generated for both the refrigeration time to first consumption (days) and the refrigeration time to last consumption (days) of products. These two distributions were implemented in @Risk for two RTE product categories: hot dogs and deli meat sliced by the manufacturer, with the latter assumed as a proxy for pre-packaged deli meats. The study also provided the percentage of individuals who consumed the whole product on the first occasion it was eaten. This percentage was used to generate a discrete distribution between time to first consumption and time to last consumption. For example, if hot dogs are the RTE product selected, the model first generates a time to first consumption and a time to last consumption of hot dogs. The model then uses the discrete distribution to select an input between these two values; the time to first consumption is chosen with a probability equal to the percentage of individuals who consumed the whole product on the first occasion it was eaten, and the time to last consumption is chosen the remainder of the time. The resulting estimate is conservative as it does not account for partial consumption of the product prior to the time to last consumption. Alternate sources either did not offer product-specific estimates or reported vague time estimates (e.g. % of products stored longer than 7 days).

*S*2.8 Temperature of Refrigerator (8. What is the temperature of the refrigerator?)

Input Values:

Deli Meat & Hot Dogs: Laplace (4.06, 2.31)

Units: °C

Source(s):[7]

Description:

A distribution of refrigeration temperatures was available from a study by Pouillot *et al*.[7], which was based on a nationally representative survey of U.S. adults (N=2,037). Refrigeration temperatures (°C) were characterized using a Laplace distribution. Although the data is not Canadian, it is assumed refrigeration temperatures are similar in both countries.

*S2.9* Counter Storage Time (9. How long is the product stored on the countertop at ambient temperature?)

Input Values:

Deli Meat & Hot Dogs: Uniform (0, 2)

Units: Hours

Source(s):[9]

Description:

The amount of time spent on the countertop prior to consumption was based on a *L. monocytogenes* consumer phase risk assessment for eggs [9]. This assessment provided distributions of ambient countertop storage time both prior to cooking and after cooking. The distribution for storage time prior to cooking was used as it is assumed that the majority of products in the model are not heated. Further, it is assumed that the consumption of reheated products occurs immediately after heating. This distribution was used in the absence of deli meat specific and hot dog specific estimates, although countertop storage time is expected to vary between products. Variation between refrigerated and frozen products is likely high due to thawing periods. However, due to a lack of data on countertop thawing practices, and the complexity of modelling microbial growth during the thawing process, it was assumed that any bacteria on frozen hot dogs do not grow during countertop storage.

*S2.10 Countertop Temperature of Product (10. What is the ambient temperature of the product in the kitchen?)*

Input Values:

Deli Meat & Hot Dogs: Uniform (Laplace (4.06, 2.31), Uniform (20, 22))

Units: °C

Source(s):[10]

Description:

Growth during countertop storage was assumed to depend on refrigerator temperature and ambient room temperature. A previous risk assessment [9] assumed countertop meat temperature varied between refrigerator temperature and room temperature. Similarly, the model estimates the countertop meat temperature using values uniformly distributed between refrigerator and room temperature. The minimum value represented the refrigerator temperature selected in each iteration of the model. The average room temperature in Canadian households, which was found to vary between 20°C and 22°C[10], was used as the maximum temperature. Thus the maximum value is represented by a uniform distribution from 20-22°C. This distribution does not account for products exposed to higher temperatures such as during storage prior to barbequing or dining outdoors. While this could underestimate microbial growth, the outdoor temperature in Canada is only >22°C for a small portion of the year.

*S2.11 Portions that Contaminate the Environment (11. Given contaminated portions, what percentage of portions contaminate the environment? (e.g. hands, kitchen, equipment))*

Input Values:

Deli Meat & Hot Dogs: Triangular (3, 26, 38)

Units: %

Source(s):[11]

Description:

The model subdivides contaminated portions based on whether cross-contamination occurs during consumer handling and preparation in the kitchen. A quantitative analysis performed by Kusumaningrum *et al*. [11] summarized the results of five separate studies which measured the prevalence of using unwashed cutting boards during food preparation. These values were used to populate a triangular distribution representing the percent of portions which contaminate the environment. For the purpose of the model, it was assumed that cross-contamination only occurs if the consumer fails to wash the contaminated cutting boards. The lowest and highest prevalence values reported were selected to represent the distribution’s minimum and maximum values. The weighted mean prevalence calculated and reported was selected to represent the distribution’s most likely value. No Canadian studies provided estimates of unwashed cutting board prevalence. The sources used however, originated in nations (e.g. U.S., Australia, U.K.) with consumers with food preparation practices that were considered comparable to Canadian consumers.

*S2.12 Transfer Rates for Contamination (12. Given cross-contamination, what percentage of CFUs on a portion will contaminate the environment?; 13. Given cross-contamination, what percentage of CFUs in the environment end up being ingested if food is a) Not Heated or b) Heated?)*

Input Values:

See table below.

Units: %

Source(s):[12]

Description:

Three *L. monocytogenes* transfer rates were used in the model: 1) transfer from a contaminated product to a cutting board, 2) transfer from a contaminated cutting board to unheated meat, and 3) transfer from a contaminated cutting board to heated meat [12]. Each of the rates in the model was defined by a discrete uniform distribution. The distribution contained six transfer rates based on various experimental conditions. The mean and standard deviation [12] were used to inform triangular distributions for each estimate. Estimates less than 0% or greater than 100% were adjusted to 0% and 100% respectively. The experimental conditions incorporated the use of two different types of cutting boards (wooden and plastic) and three different holding periods (0 seconds, 30 minutes, 1 hour). The holding period characterized the amount of time between initial contamination of the cutting board from raw chicken and the contamination of warm or cold, previously cooked chicken from the cutting board. For each iteration of the model, a transfer rate for one set of conditions (e.g. wooden cutting board and 0 second holding time) is selected, with equal probability, for each of the three aforementioned *L. monocytogenes* transfer rates. The transfer rates from cutting boards to warm and cold cooked chicken were used to represent the transfer rates to heated and unheated RTE meats respectively. The transfer rate of *L. monocytogenes* between chicken and cutting boards was assumed to be comparable to the transfer rates between RTE meats and food preparation surfaces. Although studies were available that estimated RTE meat product’s transfer rates, the focus was on processing/production facilities, and therefore the materials (e.g. stainless steel) considered were not considered as proxies for home preparation environments.

**Table S2.1.** Values for the three discrete distributions. Values represent the mean percentage of *L. monocytogenes* transferred under each set of conditions ± standard deviation.

|  | | **Holding Time** | | |
| --- | --- | --- | --- | --- |
|  | **Board Type** | *0 Seconds* | *30 Minutes* | *1 Hour* |
| **Meat to Board** | *Plastic Board* | 71.2 ± 8.3 | 72.6 ± 3.4 | 0.71 ± 4.0 |
|  | *Wooden Board* | 74.1 ± 07.8 | 62.1 ± 20.6 | 0.061 ± 18.3 |
| **Board to Cool Meat** | *Plastic Board* | 88.5 ± 06.4 | 90.2 ± 4.8 | 0.872 ± 6.9 |
|  | *Wooden Board* | 87.9 ± 6.9 | 73.5 ± 33.8 | 0.110 ± 33.0 |
| **Board to Hot Meat** | *Plastic Board* | 73.2 ± 28.5 | 51.1 ± 39.0 | 0.250 ± 37.6 |
|  | *Wooden Board* | 65.6 ± 37.9 | 21.5 ± 42.6 | 0 |

*S2.13 % of Portions Consumed Raw (14. What percentage of portions are eaten raw?)*

Input Values:

Deli Meat: 100

Hot Dogs: Uniform (0.2, Triangular (4, 7, 10))

Units: %

Source(s):[2, 8]

Description:

The model divides the contaminated hot dog portions into two preparation categories: reheated and unheated. Due to the availability of two relevant sources, a uniform distribution was used in the model. The minimum value for the distribution, 0.2%, was obtained from the results of a web-based survey of U.S. adults (N=1,212) [8]. The survey found 99.8% of households heated hot dogs before consumption; therefore the remaining households and portions are assumed to be consumed raw. The maximum value for the distribution was a triangular distribution obtained from the U.S. FDA/FSIS risk assessment [2]. Based on the results of the AMI survey and the FDA Food Safety survey, this assessment used a triangular distribution with a minimum, a mode, and a maximum of 4, 7, and 10%, respectively, to represent the percentage of hot dogs consumed raw [2]. It should be noted that in U.S. FDA/FSIS assessment, the reported triangular distribution applies only to non-frozen (refrigerated) hot dogs, as it was assumed that all frozen hot dogs would be reheated to some extent. However, in the model described herein, this triangular distribution applies to both refrigerated and frozen hot dogs as it is possible that frozen hot dogs could be consumed raw after thawing. Consumers do not typically reheat deli meat and it was assumed that 100% of deli meat portions are served raw.

*S2.14 Reheating Time (15. Factors that affect thermal inactivation: a) Reheating Time)*

Input Values:

Deli Meat: n/a

Hot Dogs: Uniform (0.5, 9.0)

Units: Minutes

Source(s):[13]

Description:

A uniform distribution was used to represent the reheating time for hot dogs. The distribution was based on the heating instructions found on commercial brand original and jumbo hot dogs [13]. These values should account for differences in both heating method, and hot dog size. There were no consumer-based studies available which identified hot dog heating times but it is assumed that the majority of people will heat hot dogs according to the manufacturer’s instructions. Consumers do not typically reheat deli meat and therefore there was no estimate included for deli meat heating time.

S*2.15 Internal Heating Temperature (15. Factors that affect thermal inactivation: b) Internal Temperature)*

Input Values:

Hot Dogs: Triangular (54, Uniform (69,73), 77)

Deli Meat: N/A

Units: °C

Source(s):[2]

Description:

A triangular distribution was used to represent the default heating temperature for hot dogs. The parameters for this distribution were obtained from the U.S. FDA/FSIS risk assessment [2]. The values used to describe internal heating temperatures of hot dogs were 54, 69-73, and 77°C, which represented the minimum temperature, a uniform distribution of most likely temperatures, and the maximum temperature, respectively. These values were based on a previous *E. coli* thermal inactivation study. In absence of a study on Canadian consumer behaviour with respect to hot dogs, the U.S. FDA/FSIS estimate, which was created by an expert panel, was utilized. It is assumed that consumers do not typically reheat deli meat; therefore no estimate was included for deli meat heating temperature.

*S2.16 Reference D, T, & Z Values (thermal inactivation)*

Input Values:

D_ref_: 0.05333333 seconds

T_ref_: 70 °C

z- value: 5.47 °C

Source(s):[14]

Description:

To calculate growth, three values were required; a reference D value, a reference T value, and a z-value. The D_ref_ value is the decimal reduction time, the heating time required to kill 90% of the organisms, at referent temperature T_ref_. The z value is the temperature required for a one log reduction in the D value. Values used in the model were obtained from Huang [14], and were specific to the thermal inactivation of *L. monocytogenes* in beef frankfurters. The D_ref_ and z values were based on the temperature of a water bath used to heat flattened 1mm thick samples of frankfurter meat. These temperatures would be a close approximation of meat temperature and therefore were comparable to the thermal inactivation at various internal temperatures of frankfurters during heating.

*S2.17 Storage Time and Temperature Prior to Retail*

Input Values:

Storage Temperature: Uniform (1, 5) °C

Storage Time: Uniform (10, 30) days

Source(s): [15]

Description:

Storage temperature prior to retail was required to calculate the lag time of *L. monocytogenes* populations. The length of storage prior to retail was required to determine the amount of lag time remaining, if any, at the retail phase. Both inputs were represented by uniform distributions [15].

### S3. Canadian Subpopulation Prevalence Data

### The section below provides a brief description of values used to characterize prevalence of at-risk subpopulations in the model. Values for each risk factor and province/territory are described. Values could not be found for all provinces/territories for each condition of interest; in these cases, descriptions are not included below and estimates for the entirety of Canada were used as surrogates.

#### S3.1 Population Size (By Province, Age, & Sex)

Source(s):[16]

Description:

Canadian national, provincial, and territorial population estimates by age and sex were obtained from the Statistics Canada CANSIM database [16]. Estimates are based on the Canadian census. Location-specific gender and age prevalence in 2014 were used to estimate the size of subpopulations based on age and comorbidities as described in the manuscript. Additionally, 1998-2014 population data were used to estimate the prevalence of risk factors when only prevalent case estimates were given; details of this are described in the respective sections below.

#### S3.2 AIDS/HIV

Source(s):[17]

Description:

National and provincial 2011 prevalent HIV/AIDS case estimates and uncertainties were obtained from a Public Health Agency of Canada report[18]. Prevalence estimates were calculated by dividing the number of prevalent cases by the 2011 population estimates from the CANSIM database [16]. The range of uncertainty was modelled using a triangular distribution with the main estimate representing the most likely value and the upper and lower uncertainty estimates representing the minimum and maximum values respectively. A combined estimate was given for the Atlantic provinces, and thus the same distribution was used for Newfoundland, Nova Scotia, and Prince Edward Island. The report did not provide estimates for the territories due to small sample sizes and large uncertainties. Therefore. the Canadian national prevalence is used if a territory is selected in the model.

#### S3.3 Cancer

Listeriosis risk factors in the Goulet *et al*. study [19] included eleven types of cancer: bladder, blood, brain, breast, ENT (ear, nose, and throat), gastrointestinal, gynecological, kidney, liver, lung, and prostate cancer. Available Canadian national, provincial, and territorial prevalence estimates by sex and cancer type were collected for the eleven cancer types. Blood cancer included Hodgkin’s lymphoma, leukemia, multiple myeloma, and non-Hodgkin lymphoma. ENT cancer included larynx, oral cavity, and pharynx cancers. No prevalence estimates were available for ear and nose cancer. Gastrointestinal cancer included colorectal, esophageal, pancreatic, and stomach cancers. Gynecological cancer included cervical, ovarian, and uterine cancers. Finally, prostate cancer is male-specific and thus no female estimates were included.

The most recent estimates identified were used. National estimates were utilized where provincial and territorial estimates were unavailable. General population estimates were used in absence of sex-specific data. Descriptions of prevalence sources can be found below. Cancer prevalence was typically determined by linking cancer incidence data with mortality data, such that anyone in the defined time period living with a previous cancer diagnosis is counted as a prevalent case. For our purposes, to determine the proportion of the population that has a weakened immune system due to cancer, this may overestimate prevalence as it could include fully recovered individuals.

##### National

Source(s):[20]

Cancer Prevalence in Source: All

Description:

National cancer prevalence was obtained from a 2012 Statistics Canada report [20]. Estimates were given as ten-year prevalence proportion (per 100,000) by cancer type, and sex. Prevalence values were divided by 100,000 prior to input into the model. The estimates did not include Québec. It is assumed that inclusion of Québec cancer prevalence would not significantly alter the national estimates.

##### Provincial/Territorial

###### Alberta

Source(s):[21-32]

Cancer Prevalence in Source: Blood (non-Hodgkin’s lymphoma and leukemia), bladder, breast, gastrointestinal (colorectal and pancreatic), gynecological (cervical and uterine), kidney, liver, lung, and prostate.

Description:

Ten-year prevalent cases were obtained from several Alberta Health Services reports[21-32]. Reports were specific to cancer type and provided an estimate of prevalent cases for both sexes combined in either 2010 or 2012. Prevalent cases were then divided by the 2010 or 2012 Alberta population respectively, which was obtained from the Statistics Canada CANSIM database [16].

###### British Columbia

Source(s):[33]

Cancer Prevalence in Source: All except liver cancer.

Description:

Lifetime prevalent cases as of 2014 for both sexes combined were obtained from the Facts & Figures page of the B.C. Cancer Agency website [33]. Prevalent cases were then divided by the 2014 British Columbia population, which was obtained from the Statistics Canada CANSIM database [16]. All time prevalence may lead to overestimation compared to other sources; however, no alternate data were found.

###### Manitoba

Source(s):[34]

Cancer Prevalence in Source: Breast, gastrointestinal (colorectal), lung, prostate.

Description:

Lifetime prevalent cancer cases were obtained from a report by Kliewar, Wajda, and Blanchard through CancerCare Manitoba [34]. The 1998 all-time prevalent case estimates for both sexes combined were used. Prevalent case estimates after 1998 were projected estimates, thus were not used. All time prevalence may lead to overestimation compared to other sources; however, no alternate Manitoba-specific prevalence data was found. Prevalent cases were then divided by the 1998 Manitoba population, which was obtained from the Statistics Canada CANSIM database [16].

###### Nova Scotia

Source(s):[35]

Cancer Prevalence in Source: Breast, gastrointestinal (colorectal), lung, prostate.

Description:

Prevalent cancer cases were obtained from a report through Cancer Care Nova Scotia [35]. Fifteen-year sex-specific prevalent cases for 2004 were used. Prevalent cases were then divided by the 2004 sex-specific Nova Scotia populations, which were obtained from the Statistics Canada CANSIM database [16].

###### Ontario

Source(s):[36]

Cancer Prevalence in Source: Breast (female only), gastrointestinal (colorectal), lung, prostate.

Description:

Ten-year, sex-specific prevalent cases for 2007 were used. Prevalent cases were then divided by the 2007 sex-specific Ontario populations, which were obtained from the Statistics Canada CANSIM database [16].

###### Prince Edward Island

Source(s):[37]

Cancer Prevalences in Source: Breast (female only), gastrointestinal (colorectal), lung, prostate.

Description:

Ten-year, sex-specific prevalent cases for 2008 were used. Prevalent cases were then divided by the 2008 sex-specific Prince Edward Island populations, which were obtained from the Statistics Canada CANSIM database[16].

###### Québec

Source(s):[38]

Cancer Prevalence in Source: All except liver cancer.

Description:

Ten-year, sex-specific prevalence estimates (per 100,000) in 1999 were used. Prevalence was divided by 100,000 prior to input into the model. Blood cancer was calculated as the sum of *lymphatic and haemopoetic tissue* and *leukemia*.

###### Saskatchewan

Source(s):[39]

Cancer Prevalence in Source: Bladder, blood, breast (female only), gastrointestinal (colorectal), gynecological (cervical and uterine), kidney (male only), prostate (male only).

Description:

Ten-year sex-specific prevalent cases in 2003 were used. The lymphoma estimate was assumed to be the sum of both Hodgkin and non-Hodgkin lymphoma cases. Lung cancer estimates also included cancer of the trachea and bronchus. Prevalent cases were then divided by the 2003 sex-specific Saskatchewan populations, which were obtained from the Statistics Canada CANSIM database [16].

###### New Brunswick, Newfoundland, Northwest Territories, Yukon & Nunavut

National estimates were used as no provincial/territorial prevalence estimates were identified.

##### *S3.4 Diabetes*

Source(s):[40]

Description:

Canadian national, provincial, and territorial diabetes prevalence estimates were obtained from the Statistics Canada CANSIM database [40] and were based on the 2013 Canadian Community Health Survey (CCHS). Prevalence estimates include Type I and Type II diabetes combined for individuals over 12 years old. It is assumed that including the children under the age of twelve years old would not significantly change the average prevalence, as it is known Type I diabetes is uncommon in children[41]. No estimate was available for Nunavut, thus the national estimate was used. Estimates for the Northwest Territories and the Yukon should be used with caution due to small sample sizes.

##### *S3.5 Dialysis*

Source(s):[42]

Description:

Prevalence was estimated by dividing the number of prevalent end-stage kidney patients using dialysis in 2013 by population sizes in 2013 from the Statistics Canada CANSIM database [16]. Dialysis included both hemodialysis and peritoneal dialysis. Combined prevalence values were provided for British Columbia and the Yukon, and for Alberta, the Northwest Territories, and Nunavut.

##### *S3.6 Heart Disease*

Source(s):[43]

Description:

Data were obtained from the Public Health Agency of Canada’s Chronic Disease Infobase[43], and was based on the 2011/12 cycle of the Canadian Community Health Survey (CCHS). National, provincial, and territorial prevalence was estimated as the number of people over twelve years old that reported having heart disease. Estimates and 95% confidence intervals were used to set up triangular distributions in the model. It is assumed that children under twelve years old do not suffer from heart disease. Therefore, the prevalence estimates were multiplied by the proportion of the population over twelve. Although the CCHS is a reputable survey, self-reporting may lead to bias in the results. Further, it is important to note that in the study by Goulet *et al*.[19], heart disease was classified as valvulopathy or those with abnormal or artificial heart valves. No such estimate was available for Canadian populations and it is assumed that the prevalence of valvulopathy is lower than self-reported heart disease. Thus risk estimates for this group may be overestimated in the model.

##### *S3.7 Inflammatory Diseases*

Crohn’s disease, giant cell arteritis, rheumatoid arthritis, and ulcerative colitis were included as inflammatory diseases in Goulet *et al*. [19]. Canadian data specific to giant cell arteritis were unavailable, and therefore this condition was excluded from the model. However, the prevalence of this condition was expected to be relatively low; the prevalence was approximately 0.075% in the United States circa 2008[44], and thus its exclusion is not expected to considerably impact the results. Crohn’s disease, rheumatoid arthritis, and ulcerative colitis prevalence estimates were identified as described below, and summed in the risk model to represent the overall prevalence of inflammatory diseases.

*S3.7.1 Rheumatoid Arthritis*

###### National

Source(s):[45]

Description:

The model used prevalent rheumatoid arthritis cases for the Canadian population in 2010. The data were obtained from a 2011 report by the Arthritis Alliance of Canada [45]. The prevalence was estimated by a workshop of experts aiming to describe the current and future epidemiology and burden of arthritis in Canada. Prevalent cases were then divided by the 2010 Canadian population, which was obtained from the Statistics Canada CANSIM database [16].

###### British Columbia

Source(s):[46]

Description:

Estimates were based on prevalent cases of rheumatoid arthritis in British Columbia in 2000 derived from administrative billing data [46]. Cases were then divided by the 2000 British Columbia population, which was obtained from the Statistics Canada CANSIM database [16].

###### Ontario

Source(s):[47]

Description:

Estimates were based on 2010 prevalent cases of rheumatoid arthritis in Ontario. These cases were identified through the Rheumatoid Arthritis Administrative Database [47]. Cases were then divided by the 2010 Ontario population, which was obtained from the Statistics Canada CANSIM database [16].

###### Québec

Source(s):[48]

Description:

Rheumatoid arthritis prevalence estimates for Québec in 2008 were obtained from a study using physician billing and hospital administration data[48].

*S3.7.2 Crohn’s and Ulcerative Colitis*

Source(s):[49]

Description:

Prevalence estimates were based on prevalent cases of Crohn’s and ulcerative colitis in Canadian, provincial, and territorial populations in 2008. The prevalence was estimated using both data from the CCHS and a database study funded by the Crohn’s and Colitis Foundation of Canada [49]. Prevalent cases were divided by the 2008 respective Canadian, provincial, and territorial populations, which were obtained from the Statistics Canada CANSIM database[16]. A combined prevalence was used for the territories as cases were pooled in the report.

##### *S3.8 Liver Disease*

Liver disease was estimated as the combined prevalence of Hepatitis A, B, and C. The Goulet *et al*. study [19]used the prevalence of cirrhosis to represent liver disease; however, no Canadian prevalence estimate of cirrhosis was available. Hepatitis infection is one cause of cirrhosis/liver disease but hepatitis does not always cause cirrhosis, nor is it the only cause of liver disease. Prevalence of each cause of liver disease (e.g. alcoholism) was not included as this might overestimate the prevalence of cirrhosis/liver disease.

*S3.8.1 Hepatitis A*

Source(s):[50]

Description:

There were no data on the number of prevalent Hepatitis A cases in Canada. Instead, the number of incident cases for Canada in 2012 was obtained from PHAC’s Notifiable Diseases Online database [50]. This value was divided by the 2012 Canadian population from the Statistics Canada CANSIM database [16]. The value should be updated if prevalent case data becomes available, as Hepatitis A incidence likely underestimates prevalence.

*S3.8.2 Hepatitis B*

Source(s):[51]

Description:

The estimated seroprevalence of Hepatitis B infections in Canada along with 95% confidence intervals were used to set up a triangular distribution in the model. There was no provincial prevalence data available.

*S3.8.3 Hepatitis C*

Source(s):[52]

Description:

Hepatitis C prevalence estimates in 2007 for Canada, the provinces, and the territories were used and based on models using various exposure categories and places of birth.

##### *S3.9 Pregnancy*

Source(s):[16, 53, 54]

Description:

Prevalence of pregnancy was determined using the Statistics Canada CANSIM database and a report from the Canadian Institute for Health Information. Estimates were determined by first summing live births[54], fetal loss[54] and abortions for 2011[53]. This value was then divided by the 2011 population [16]. To account for the length of pregnancy, 9 months, the value was finally multiplied by 0.75. National, provincial, and territorial estimates were calculated.

##### *S3.10 Transplants*

Source(s): [42]

Description:

Overall transplant prevalence was calculated by summing the prevalence of heart, intestinal, kidney, liver, lung, and pancreas transplants. Prevalence was estimated by dividing the number of prevalent transplant cases by population sizes in 2013 from the Statistics Canada CANSIM database [16]. National and provincial prevalence data were available for the majority of transplant types. Intestinal and pancreas transplant prevalence was unavailable. Instead, national estimates for the number of transplants from 2004-2013 were used. In some cases, the number of transplants was recorded as “<5”; it was assumed four transplant cases had occurred.

### REFERENCES

1. **Ross T*, et al*.** *Listeria monocytogenes* in Australian processed meat products: risks and their management: Final report. 2009.

2. **U.S. Food and Drug Administration, Center for Food Safety and Applied Nutrition, U.S. Department of Agriculture, Food Safety and Inspection Service, and Centers for Disease Control and Prevention.** Quantitative assessment of the relative risk to public health from food-borne *Listeria monocytogenes* among selected categories of ready-to-eat foods. 2003. (http://www.fda.gov/downloads/Food/FoodScienceResearch/UCM197330.pdf) Accessed 27 September 2015.

3. **Sheehy T, Roache C, Sharma S.** Eating habits of a population undergoing a rapid dietary transition: portion sizes of traditional and non-traditional foods and beverages consumed by Inuit adults in Nunavut, Canada. *Nutrition Journal J* 2013; 1**2**: 1-11.

4. **Pouillot R*, et al*.** Quantitative Risk Assessment of *Listeria monocytogenes* in French Cold‐Smoked Salmon: I. Quantitative Exposure Assessment. *Risk Analysis* 2007; **27**: 683-700.

5. **EcoSure.** 2007 United States cold temperature evaluation design and summary pages. 2008. (http://foodrisk.org/exclusives/EcoSure/). Accessed 27 September 2015.

6. **Canadian Food Inspection Agency.** Guide to food safety. 2010. (http://www.inspection.gc.ca/food/non-federally-registered/safe-food-production/guide/eng/1352824546303/1352824822033). Accessed 27 September 2015.

7. **Pouillot R*, et al*.** Estimating parametric distributions of storage time and temperature of ready-to-eat foods for U.S. households. *Journal of Food Protection* 2010; **73**: 312-321.

8. **Cates SC*, et al*.** Consumer knowledge, storage, and handling practices regarding *Listeria* in frankfurters and deli meats: results of a Web-based survey. *Journal of Food Protection* 2006; **69**: 1630-1639.

9. **Mokhtari A*, et al*.** Consumer-phase *Salmonella enterica* serovar Enteritidis risk assessment for egg-containing food products. *Risk Analysis* 2006; **26:** 753-768.

10. **Dewis G.** *Controlling the temperature in Canadian homes:* Statistics Canada, 2008. (Environment accounts and statistics technical paper series; catalogue no. 16-001-M, no. 6.)

11. **Kusumaningrum HD*, et al*.** A quantitative analysis of cross-contamination of *Salmonella* and *Campylobacter* spp. via domestic kitchen surfaces. *Journal of Food Protection* 2004; **67**: 1892-1903.

12. **Goh SG*, et al*.** Transmission of *Listeria monocytogenes* from raw chicken meat to cooked chicken meat through cutting boards. *Food Control* 2014; **37**: 51-55.

13. **Farmland.** Hickory smoked hot dogs - original (<http://www.farmlandfoods.com/products/hickory-smoked-hot-dogs-original.html>). Accessed 27 September 2015.

14. **Huang L.** Computer-controlled microwave heating to in-package pasteurize beef frankfurters for elimination of *Listeria monocytogenes*. *Journal of Food Process Engineering* 2005; **28**: 453-477.

15. **Pradhan AK*, et al*.** Quantitative risk assessment for *Listeria monocytogenes* in selected categories of deli meats: impact of lactate and diacetate on listeriosis cases and deaths. *Journal of Food Protection* 2009; **72**: 978-989.

16. **Statistics Canada.** *Table 051-0001 - Estimates of population, by age group and sex for July 1, Canada, provinces and territories, annual (persons unless otherwise noted)*: CANSIM (database). Accessed 27 September 2015.

17. **Public Health Agency of Canada.** Chapter 1: National HIV prevalence and incidence estimates for 2011. 2014. (http://www.phac-aspc.gc.ca/aids-sida/publication/epi/2010/1-eng.php). Accessed 27 September 2015.

18. **Public Health Agency of Canada.** HIV/AIDS Epi Updates: National HIV Prevalence and Incidence Estimates for 2011. 2014. (http://www.phac-aspc.gc.ca/aids-sida/publication/epi/2010/1-eng.php). Accessed 27 September 2015.

19. **Goulet V*, et al*.** Incidence of listeriosis and related mortality among groups at risk of acquiring listeriosis. *Clinical Infectious Diseases* 2012; **54**: 652-660.

20. **Ellison LF, Wilkins K.** *Canadian trends in cancer prevalence*: Statistics Canada, 2012. (http://www.statcan.gc.ca/pub/82-003-x/2012001/article/11616-eng.htm) Accessed 27 September 2015.

21. **Cancer Care.** *Cancer surveillance: 2010 report on cancer statistics in Alberta - Liver Cancer*: Alberta Health Services, 2012. (http://www.albertahealthservices.ca/poph/hi-poph-surv-cancer-liver-2010.pdf). Accessed 27 September 2015.

22. **Cancer Care**. *Cancer surveillance: 2010 report on cancer statistics in Alberta - Cervical Cancer:* Alberta Health Services, 2012. (http://www.albertahealthservices.ca/poph/hi-poph-surv-cancer-cervical-2010.pdf) Accessed 27 September 2015.

23. **Cancer Care.** *Cancer surveillance: 2010 report on cancer statistics in Alberta - Pancreatic Cancer:* Alberta Health Services, 2012. (http://www.albertahealthservices.ca/poph/hi-poph-surv-cancer-pancreas-2010.pdf) Accessed 27 September 2015.

24. **Cancer Control AB**. *Surveillance & Reporting: 2012 Report on cancer statistics in Alberta - Prostate Cancer:* Alberta Health Services, 2015. (http://www.albertahealthservices.ca/assets/healthinfo/poph/hi-poph-surv-cancer-cancer-in-alberta-2012.pdf). Accessed 27 September 2015.

25. **Cancer Control AB.** *Surveillance & Reporting: 2012 Report on cancer statistics in Alberta - Kidney Cancer:* Alberta Health Services, 2015. (http://www.albertahealthservices.ca/assets/healthinfo/poph/hi-poph-surv-cancer-kidney-2012.pdf). Accessed 27 September 2015.

26. **Cancer Control AB**. *Surveillance & Reporting: 2012 Report on cancer statistics in Alberta - Lung Cancer:* Alberta Health Services, 2015.( http://www.albertahealthservices.ca/assets/healthinfo/poph/hi-poph-surv-cancer-lung-2012.pdf). Accessed 27 September 2015.

27. **Cancer Control AB.** *Surveillance & Reporting: 2012 Report on cancer statistics in Alberta - Colorectal Cancer:* Alberta Health Services, 2015.(http://www.albertahealthservices.ca/assets/healthinfo/poph/hi-poph-surv-cancer-colorectal-2012.pdf) . Accessed 27 September 2015.

28. **Cancer Control AB.** *Surveillance & Reporting: 2012 Report on cancer statistics in Alberta - Breast Cancer:* Alberta Health Services, 2015.( http://www.albertahealthservices.ca/assets/healthinfo/poph/hi-poph-surv-cancer-breast-2012.pdf). Accessed 27 September 2015.

29. **Cancer Control AB**. *Surveillance & Reporting: 2012 Report on cancer statistics in Alberta - Non-Hodgkin Lymphoma:* Alberta Health Services, 2015. (http://www.albertahealthservices.ca/assets/healthinfo/poph/hi-poph-surv-cancer-nhl-2012.pdf). Accessed 27 September 2015.

30. **Cancer Control AB.** *Surveillance & Reporting: 2012 Report on cancer statistics in Alberta - Bladder Cancer:* Alberta Health Services, 2015. (http://www.albertahealthservices.ca/assets/healthinfo/poph/hi-poph-surv-cancer-bladder-2012.pdf). Accessed 27 September 2015.

31. **Cancer Control AB.** *Surveillance & Reporting: 2012 Report on cancer statistics in Alberta - Uterine Cancer:* Alberta Health Services, 2015. ( http://www.albertahealthservices.ca/assets/healthinfo/poph/hi-poph-surv-cancer-uterine-2012.pdf). Accessed 27 September 2015.

32. **Cancer Control AB**. *Surveillance & Reporting: 2012 Report on cancer statistics in Alberta - Leukemia:* Alberta Health Services, 2015. (http://www.albertahealthservices.ca/assets/healthinfo/poph/hi-poph-surv-cancer-leukemia-2012.pdf). Accessed 27 September 2015.

33. **BC Cancer Agency.** Number of prevalent cancers by cancer type and year (2005-2020). 2015. (<http://www.bccancer.bc.ca/health-info/disease-system-statistics/bc-cancer-statistics/facts-and-figures>). Accessed 27 September 2015.

34. **Kliewer E, Wajda A, Blanchard J.** *The increasing cancer burden: Manitoba cancer projections 1999-2025:* CancerCare Manitoba, 2001. (http://www.cancercare.mb.ca/cancercare_resources/EPI/pdfs/1999-2025projections.pdf). Accessed 27 September 2015.

35. **Saint-Jacques N*, et al*.** *Understanding Cancer in Nova Scotia: A statistical report by Cancer Care Nova Scotia with a focus on 2000-2004*: Surveillance and Epidemiology Unit, Cancer Care Nova Scotia, 2006. (http://www.cancercare.ns.ca/site-cc/media/cancercare/understandingcancer.pdf). Accessed 27 September 2015.

36**. Bahl S*, et al*.** *Cancer in Ontario: Overview:* Cancer Care Ontario, 2010. (https://www.cancercare.on.ca/common/pages/UserFile.aspx?fileId=81843). Accessed 27 September 2015.

37. **Morrison H*, et al*.** *Prince Edward Island Cancer Trends: 1980-2009*: Prince Edward Island Department of Health and Wellness, 2012. (http://www.gov.pe.ca/photos/original/CancerTrends09.pdf). Accessed 27 September 2015.

38. **Louchini R*, et al*.** Trends in cancer prevalence in Quebec. *Chronic Diseases in Canada* 2006; **27**: 110-119.

39. **Alvi R, Tonita J.** *Saskatchewan cancer control report: profiling cancer prevalence, 1984-2003.* Saskatchewan Cancer Agency, 2008. (http://www.saskcancer.ca/adx/aspx/adxGetMedia.aspx?DocID=94,5,1,Documents&MediaID=ef1a377f-201c-48f7-b197-0b8374da99bc&Filename=SCCR+2007.pdf). Accessed 27 September 2015.

40. **Statistics Canada**. *Table 105-0501 - Health indicator profile, annual estimates, by age group and sex, Canada, provinces, territories, health regions (2013 boundaries) and peer groups, occasional:* CANSIM (database). Accessed 27 September 2015.

41. **Public Health Agency of Canada.** Diabetes in Canada: Facts and figures from a public health perspective. 2011. (http://www.phac-aspc.gc.ca/cd-mc/publications/diabetes-diabete/facts-figures-faits-chiffres-2011/index-eng.php) Accessed 27 September 2015.

42**. Webster G*, et al*.** *Canadian Organ Replacement Register Annual Report: Treatment of End-Stage Organ Failure in Canada 2004–2013.* Canadian Institute for Health Information*,* 2015.

43. **Centre for Chronic Disease Prevention, Public Health Agency of Canada.** Chronic Disease and Injury Indicator Framework, 2014 Edition. 2014. (http://infobase.phac-aspc.gc.ca/cdif/) Accessed 27 September 2015.

44. **Lawrence RC*, et al*.** Estimates of the prevalence of arthritis and other rheumatic conditions in the United States: Part II. *Arthritis & Rheumatism* 2008; **58**: 26-35.

45. **Bombardier C, Hawker G, Mosher D.** *The impact of arthritis in Canada: today and over the next 30 years:* Arthritis Alliance of Canada, 2011. (http://www.arthritisalliance.ca/images/PDF/eng/Initiatives/20111022_2200_impact_of_arthritis.pdf). Accessed 27 September 2015.

46. **Lacaille D*, et al*.** Gaps in care for rheumatoid arthritis: a population study. *Arthritis Care & Research* 2005; **53**: 241-248.

47. **Widdifield J*, et al*.** The epidemiology of rheumatoid arthritis in Ontario, Canada. *Arthritis & Rheumatology* 2014; **66**: 786-793.

48. **Bernatsky S*, et al***. Rheumatoid arthritis prevalence in Quebec. *BMC research notes* 2014; **7**: 937.

49. **Crohn’s and Colitis Foundation of Canada.** The burden of Inflammatory Bowel Disease (IBD) in Canada. 2008. (http://www.ccfc.ca/atf/cf/%7B282e45d9-a03a-49d1-883c-39f4feaf7246%7D/BIBDC%20FINAL%20OCTOBER%2029TH%20EN.PDF). Accessed 27 September 2015.

50. **Public Health Agency of Canada***. Hepatitis A*: Notifiable Diseases Online (database), 2012. (http://dsol-smed.phac-aspc.gc.ca/dsol-smed/ndis/index-eng.php ) Accessed 27 September 2015.

51. **Rotermann M*, et al*.** Seroprevalence of hepatitis B and C virus infections: results from the 2007 to 2009 and 2009 to 2011 Canadian Health Measures Survey. *Statistics Canada Health Reports* 2013; **24**: 3-13.

52. **Remis RS.** *Modelling the incidence and prevalence of hepatitis C infection and its sequelae in Canada, 2007:* Health Canada, 2007.( http://www.phac-aspc.gc.ca/sti-its-surv-epi/model/pdf/model07-eng.pdf) Accessed 27 September 2015.

53. **Canadian Institute for Health Information.** Induced abortions reported in Canada in 2011. 2014. (https://www.cihi.ca/sites/default/files/document/ta_11_alldatatableS30130221_en.pdf). Accessed 27 September 2015.

54. **Statistics Canada**. *Table 102-4515 - Live births and fetal deaths (stillbirths), by type (single or multiple), Canada, provinces and territories, annual (number)*: CANSIM (database) : Accessed 27 September 2015.

1. Conversion formula: °C = (°F – 32) / 1.8 [↑](#footnote-ref-1)
2. Conversion formula: °C = (°F – 32) / 1.8 [↑](#footnote-ref-2)
